# Professional identity of medical students: Proposing a Meta Static Structural Model

**DOI:** 10.30476/JAMP.2021.89121.1364

**Published:** 2021-10

**Authors:** LEILA AFSHAR, SHAHRAM YAZDANI, HOMA SADEGHI AVVAL SHAHR

**Affiliations:** 1 Department of Medical Ethics, Shahid Beheshti University of Medical Sciences, Tehran, Iran; 2 Virtual School of Medical Education and Management, Shahid Beheshti University of Medical Sciences, Tehran, Iran

**Keywords:** Professional identity, Professionalism, Medical students, Social identification, Model

## Abstract

**Introduction:**

The realization of professionalism and its desirable consequences, as the most important goal of medical education, primarily depends on identifying the process and mechanism of the professional identity formation, which in turn requires the accurate identification of components and structure of the concept of professional identity. Therefore, the aim of this study was to synthesize a static structural model for professional identity, based on the results of our previous research.

**Methods:**

In this study the theory or model construction methodology was used to synthesize a static structural model of professional identity formation for medical students. In this regard, the Walker and Avant method was followed through three steps: specifying focal concepts, reviewing the literature, and organizing concepts into an integrated and efficient representation.

**Results:**

In this study, based on the analysis of 9 selected conceptual models in the field of socialization and professional identity, first the key concepts of each model were extracted and then by carefully examining these concepts and determining their relationships and reviewing related texts, dimensions and components of professional identity were determined and presented in the form of a comprehensive structural static model.

**Conclusion:**

The advantage of the proposed model over the existing models is the explicit presentation of the dimensions, constructs, and sub-constructs of the concept of professional identity. In addition, this model can be used as a general pattern in all non-medical professions.

## Introduction

 Professional identity formation in medical students, as the most important goal of medical education, and the path to the realization of professionalism ( 1 ), has been the focus of researchers in this field for about two decades. In this regard, many conceptual models and frameworks have been developed, and efforts have been made to determine the nature and components of professional identity, how it is formed, and the factors influencing its formation ( 2 - 16 ). 

Although these studies have played an important role in identifying the complex concept of professional identity, and suggesting several educational interventions to facilitate its formation, professionalism, as the main expected outcome of these interventions, has not yet been revealed.

Perhaps the cause of these failures can be found in the special features of this process, which are mentioned below:

• The professional identity formation is a complex and multi-layered process that requires the internalization of a wide range of values, behaviors, and perceptions that are often implicit, and formed through authentic work experiences sub-consciously ( 17 , 18 ).
• This process occurs simultaneously both at the individual level (psychological adaptation) and at the collective level (achieving acceptance and full participation in the professional community) ( 9 ), and therefore, unlike its visible external dimensions, examining and measuring the internal dimensions are not an easy task.
• Personal identity is the foundation of professional identity ( 19 ), and based on the Kegan model, individuals are not the same in terms of the stages of social maturity ( 20 ); therefore, the speed and ease of professional socialization process, and identity formation will be different in peers.
• The influence of uncontrollable factors such as genetics, gender, and age on the process of socialization and the formation of professional identity ( 21 ), causes people to show different perceptions, and reactions in the face of the same experiences.
• Several psychological factors affect how a professional identity develops, and it is not clear that how the desired image of professional- self in the future can affect professional identity development ( 17 ).
• Perceived incompatibility between personal identity, and the stereotypical characteristics of members of the profession may weaken people's understanding of the practicality of the career path and the motivation to pursue it ( 22 , 23 ).

Given these complexities, it is not possible to propose effective interventions without a deep and comprehensive understanding of this process. The more accurate the understanding of this process, the more effective the proposed interventions will be.

In this regard, in our previous study, we proposed an analytic definition of professional socialization ( 24 ), as a fundamental process through which the professional identity of medical students is formed.

According to this definition, professional socialization is a non-linear, continuous, interactive, personal and psychosocial process that is formed through the internalization of the specific culture of the professional community and its main outcome is the formation of professional identity ( 24 ). 

In the next step, it is necessary to identify the dimensions and components of the concept of professional identity, and to propose a conceptual model for it as a basis for monitoring the development of its components during the socialization process.

Although there are several conceptual models about professional identity in the literature, each of them often deals with only one dimension of this concept and from a specific perspective.

Therefore, the purpose of the present study is designing a comprehensive static structural model for professional identity. By identifying the components and subcomponents of this concept, it is possible to follow how they evolve in the process of socialization, and as a result, opportunity to intervene and facilitate the formation of a desirable professional identity of medical students would be possible.

## Methods

 In this study, a theory or model construction method was used to synthesize a comprehensive static structural model for professional identity for medical students. In this regard, Walker and Avant approach to model synthesis was followed. According to Walker and Avant ( 25 ), the following three steps was used to synthesize the model:

*Step 1: “Specifying focal concepts to serve as anchors for the synthesized theory”.*

The theorist must first choose the subject to synthesize the theory, and to do this, s/he defines a focal concept and moves from the focal concept to other related concepts. In this stage of the study, the phrase “professional identity formation” was selected as a focal concept. 

*Step 2: “Reviewing literature to identify factors related to the focal concepts and to specify the nature of relationships”:*

In the next step, a detailed search and review of the literature until 2017, in the main databases: EBSCO CINAHL scientific databases EBSCO CINAHL, Web of Science, Eric, Pub Med, Scopus, and Google Scholar was performed to achieve existing conceptual models and frameworks related to the focal concept of “professional identity formation”.

Only articles containing a conceptual model or framework, published in English, and with free access to the entire article were included in the review, and quantitative or experimental articles were excluded. In this way, we reached 9 conceptual models which were carefully examined.

*Step 3: “Organizing concepts and statements into an integrated and efficient representation of the phenomena of interest.”*

After collecting data from different information sources, the theorist should depict the complex relationships between the concepts related to the phenomenon of interest, and organize the general pattern of relationships between them ( 25 ).

One of the mechanisms for developing a theory or theoretical framework is the collapse of several very similar concepts into a more comprehensive summary concept ( 25 ), then placing more relevant concepts in the main “blocks”, and finally determining their interrelationships.

In this way, the concepts obtained from the previous steps as fragmented knowledge were integrated into a comprehensive model.

## Results

In the first step of the study, the concept of “professional identity formation” was selected as the focal concept of interest. While many studies have been published on professional identity formation, none of them has addressed the structure and components of this concept through a comprehensive approach. It seems necessary to identify the components and elements of professional identity as the first step to draw a detailed roadmap to monitor how these elements evolve during the process of socialization and management.

**Figure 1:**
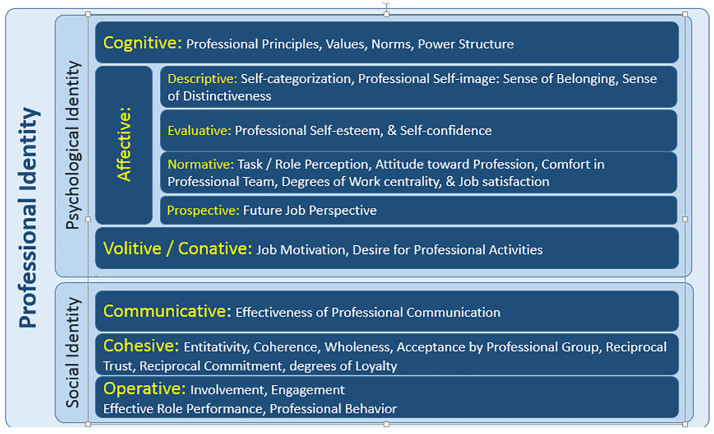
Psychosocial Model of Professional Identity (PMPI)

In the second step, 9 conceptual models obtained from this search were carefully examined in terms of the type and focus of the model, and the proposed results in relation to the concepts of “professional identity formation” (Table 1); then, the key concepts and components of each model in relation to “professional identity formation” were extracted (Table 2). In the third step, similar concepts were categorized into two main dimensions: psychosocial and social. Then, by in-depth study of the concepts of each dimension and the relevant literature, during a precise intellectual inductive process, the concepts of each dimension were divided into subgroups, the more relevant concepts being organized as defined "blocks" and their interrelationships being determined (Table 3). It is worth noting that the idea for part of this division (prospective and normative subcomponents of affective realm) stemmed from Kelchtermans' proposed concepts for teacher's professional development ( 26 ). Thereupon, the comprehensive model of Psychosocial Model of professional Identity (PMPI) was developed ( Figure1).

**Table 1:** Selected models of socialization and professional identity

Title	First author/ Year of publication	Kind of model	Focus of model	Outcomes
Exploring the transition of undergraduate medical students into a clinical clerkship using organizational socialization theory	Atherley, A.E./2016 ( 14 )	Descriptive- prescriptive	The process of transition to clinical training	Describing the factors influencing this process and proposing recommendations to facilitate it
The professionalization of medical students: A longitudinal analysis of professional identity formation and professionalism perceptions in second and third year medical students	Byram, J.N./ 2017 ( 16 )	Descriptive- normative	The process of professional identity formation & medical students' perceptions of professionalism and its impact on their professional identity formation	Main themes of Professional Identity Formation: (1) Connecting to Image of Medicine, (2) Exploring Self in Medicine, (3) Embodying Role in Medicine, (4) Exploring Specialty Choice, and (5) Internalizing of Professional Values and Characteristics
A schematic representation of the professional identity formation and socialization of medical students and residents: A guide for medical educators	Cruess, R. L./2015 ( 11 )	Explanatory/Causal	Factors influencing professional identity formation, emphasizing the role of faculty & active role of student	Describing various elements of socialization process (Knowledge Acquisition
				Explaining the mechanisms
				that influences the socialization process (internally & externally)
Experiences in becoming a paramedic: The professional socialization of university qualified paramedics	Devenish, A./2016 ( 12 )	Descriptive-normative	Stages of professional socialization	Setting 4 stages for professional socialization and describing the events and characteristics of each stage
Professional Socialization in Nursing	Eden’s, G./1987 ( 2 )	Explanatory/Causal	Affecting factors & Outcomes of the socialization process.	Domains of potential professional self-growth (Self-image, role concept, attitudes, values, and personality as) as outcomes of the socialization process.
A conceptual framework for the professional socialization of social workers	Miller, S.E./ 2010 ( 5 )	Descriptive	Stages of professional socialization	Describing the stages of professional socialization and the contents of each stages
A conceptual model of professional socialization within student affairs graduate preparation programs	Perez, R. J./2016 ( 13 )	Explanatory	Cognitive mechanisms involved in the socialization process	Explaining the dynamic interaction between people and the environment in the process of socialization with an emphasis on the sense-making and self-authoring frameworks
Undergraduate socialization: A conceptual approach	Weidman, J.C./ 1989 ( 3 )	Explanatory/Causal	College impact & Non-cognitive outcome of socialization process	Categorizing of affecting factors on career choice, and life style preferences as:
				Students backgrounds, parental socialization, non-college reference groups, & college experiences (normative context and socialization process).
Socialization of graduate and professional students in higher education: A perilous passage?	Weidman, J. C./2001 ( 4 )	Descriptive	Elements of socialization process in each stage of professional socialization	Investment, Involvement) & setting interactive stages for professional socialization (Anticipatory, Formal, Informal, Personal)

**Table 2:** The main components of professional identity extracted from the selected models

Selected model titles	First author/ Year of publication	Key components of each model
Exploring the transition of undergraduate medical students into a clinical clerkship using organizational socialization theory	Atherley, A.E./ 2016 ( 14 )	Self-efficacy, Internal motivation, Role clarity, performance, personal development, professional development
The professionalization of medical students: A longitudinal analysis of professional identity formation and professionalism perceptions in second- and third-year medical students	Byram, J.N./2017 ( 16 )	Doubting, Challenging, Confirming, Adapting, Accommodating, Refusing, Impersonating, Emulating, Practicing, Communicating, Evaluating, Participating, Perceiving, Imitating, Envisioning, Selecting & Reinforcing & Enriching & Prioritizing professional values, Detaching, Using medical terms
A schematic representation of the professional identity formation and socialization of medical students and residents: A guide for medical educators	Cruess, R./2015 ( 11 )	Learning to live with ambiguity, Learning the hierarchy & power relationships, Learning the Symbols & rituals, Learning detached concern, Learning medical Language, Learning to play the role, Self-assessment, Increased competence, Unconscious reflection, Marginalization, Participation, Social interaction
Experiences in becoming a paramedic: The professional socialization of university qualified paramedics	Devenish, A./2016 ( 12 )	Adjusting to the culture, Biculturalism. Building confidence, Stereotypical role image, Understanding the role, Marginalization, Gaining Acceptance, Increased level of acceptance, Focusing on skills
Professional Socialization in Nursing	Edens, G./1987 ( 2 )	Values, Attitude, Self-image, Role conception, cue consistency, personality
A conceptual framework for the professional socialization of social workers.	Miller, S.E./2010 ( 5 )	Values, Attitudes, Norms, Culture, Interpersonal relationship, Power structure, Engagement
A conceptual model of professional socialization within student affairs graduate preparation programs	Perez, R.J./2016 ( 13 )	Values, Meaning making, Role expectation, Assumption about profession, Increased capacity for self-authorship
Undergraduate socialization: A conceptual approach	Weidman, J.C./ 1989 ( 3 )	Values, Intrapersonal process, Career choice, Integration, Interaction
Socialization of graduate and professional students in higher education: A perilous passage?	Weidman, J.C./2001 ( 4 )	Learning Institutional culture, Peer climate, Integration, Interaction, Involvement, investment, Commitment

**Table 3:** Classification of concepts extracted from the selected models

The main Conceptual Dimensions	Extracted and Main Concepts	Relevant Conceptual Domain
Psychological dimension	Values: Edens, G./1987 ( 2 ); Miller, S.E./2010 ( 5 ); Perez R.J./2016 ( 13 ); &Weidman J.C./ 1989 ( 3 ), Learning to live with ambiguity: Cruess, R./2015( 11 ), Learning the hierarchy & power relationships: Cruess, R./2015 ( 11 ), Learning the Symbols & rituals: Cruess, R./2015 ( 11 ), Adjusting to the culture: Devenish, A./2016 ( 12 ), Biculturalism: Devenish, A./2016 ( 12 ), Meaning making: Perez, R.J./2016 ( 13 ), Selecting & Reinforcing & Enriching & Prioritizing professional values: Byram, J.N./2017 ( 16 )	Cognitive
	Unconscious reflection: Cruess, R./2015 ( 11 )
	Professional Principles, Values, Norms, Power Structure
	Self-image: Edens, G./1987 ( 2 ), Intrapersonal process: Weidman, J.C./ 1989 ( 3 ), Self-assessment: Cruess, R./2015 ( 11 ), Increased competence: Cruess, R./2015 ( 11 ), Building confidence: Devenish, A./2016 ( 12 ), Increased capacity for self-authorship: Perez, R.J./2016 ( 13 ), Self-efficacy: Atherley, A.E./ 2016 ( 14 ), Socialization negotiation: Cruess, R./2015 ( 11 ), Doubting about place in medicine: Byram, J.N./2017 ( 16 ), Reexamine commitment: Byram, J.N./2017 ( 16 ), Reaffirm commitment: Byram, J.N./2017 ( 16 ), Attitude: Edens, G./1987 ( 2 ); Miller, S.E./2010 ( 5 ), Role conception: Edens, G./1987 ( 2 ), Detached concern: Cruess, R./2015 ( 11 ), Loss of innocence: Cruess, R./2015 ( 11 ), Cynicism: Cruess, R./2015 ( 11 ), Situational adaptation: Miller, S.E./2010 ( 5 ), Stereotypical role image: Devenish, A./2016 ( 12 ), Deep understanding of the role: Devenish, A./2016 ( 12 ), Assumption about profession: Perez, R.J./2016 ( 13 ), Accurate role expectation: Perez, R.J./2016 ( 13 ), Role clarity: Atherley, A.E./ 2016 ( 14 ), Investment: Weidman, J.C./2001 ( 4 ), Specialties: Byram, J.N./2017 ( 16 ), Envisioning self as a member of specialties: Byram, J.N./2017 ( 16 ), Evaluate self in future specialty: Byram, J.N./2017 ( 16 ), Individual fit with specialty: Byram, J.N./2017 ( 16 )	Affective
	Self-categorization, Professional Self-image: Sense of Belonging, Sense of Distinctiveness, Professional Self-esteem, & Self-confidence, Task / Role Perception, Attitude toward Profession, Comfort in Professional Team, Degrees of Work centrality, & Job satisfaction, Future Job Perspective	
	Career choice: Weidman, J.C./ 1989 ( 3 ), Helping people: Devenish, A./2016 ( 12 ), Choosing an employer: Devenish, A./2016 ( 12 ), Internal motivation: Atherley, A.E./ 2016 ( 14 ), Selecting specialty: Byram, J.N./2017 ( 16 )	Volitive
	Job Motivation, Desire for Professional Activities
Social domain	Language: Cruess, R./2015 ( 11 ), Marginalization: Devenish, A./2016 ( 12 ), Communication: Byram, J.N./2017 ( 16 ), Using medical terms Byram, J.N./2017 ( 16 ), Convey medical knowledge to others: Byram, J.N./2017 ( 16 ), Interaction: Weidman, J.C./ 1989 ( 3 ) & Weidman, J.C./2001 ( 4 ), Interact with patients: Byram, J.N./2017 ( 16 )	Communicative
	Effectiveness of Professional Communication	
	Integration: Weidman, J.C./ 1989 ( 3 ) & Weidman, J.C./2001 ( 4 ), Participation: Cruess, R./2015 ( 11 ), Gaining acceptance: Devenish, A./2016 ( 12 ), Increased level of acceptance: Devenish, A./2016 ( 12 ), Increased responsibility: Devenish, A./2016 ( 12 )	Cohesive
	Coherence, Wholeness, Acceptance by Professional Group, Reciprocal Trust, Reciprocal Commitment, degrees of Loyalty	
	Involvement: Weidman, J.C./2001 ( 4 ), Social interaction: Cruess, R./2015 ( 11 ), Clinical/nonclinical experiences: Cruess, R./2015 ( 11 ), Focusing on skills Devenish, A./2016 ( 12 ), Academic performance: Perez, R.J./2016 ( 13 ) & Atherley, A.E./ 2016 ( 14 ), Relationship building: Atherley, A.E./ 2016 ( 14 ), Impersonating: Byram, J.N./2017 ( 16 ), Emulating: Byram, J.N./2017 ( 16 ), Practicing: Byram, J.N./2017 ( 16 )	Operative
	Involvement, Engagement, Effective Role Performance, Professional Behavior	

### Psychosocial Model of professional Identity (PMPI)

According to the proposed model of this study (PMPI), professional identity consists of two dimensions, psychological and social, each with several domains.

### Psychological Dimension

The psychological dimension of professional identity was divided into three parts: cognitive, affective, and volitive.

**Cognitive Realm:** This part itself includes *professional principles, values, norms, and power structure.*

Kwon, et al. quoting Bowen and Schneider ( 27 ), contend that values and norms, as important components of any professional's culture, guide and control the activities and behavior of members of a professional group, and are closely related to each other. Professional values are intentionally chosen by the profession to shape the identity, principles and beliefs of the group members ( 28 ).

In professional socialization, people who enter a new profession, while having their own personal values, learn and internalize the values of the new profession through observation of role models ( 29 ).

The formation of a proper professional identity depends on the integration of personal and professional values, and commitment to both of these values ( 11 ).

Another cognitive component of the professional identity is the understanding and acceptance of the hierarchical structure of power in medicine. In the socialization process, physicians learn from the very beginning of entering a medical school to respect the hierarchy of power, and avoid challenging the power and authority of senior physicians ( 30 , 31 ).

**Affective Realm:** The affective realm includes *descriptive, evaluative, normative, and prospective* components.

***Descriptive component*** includes professional *self-categorization, professional self-image, sense of belonging and a sense of distinction.*

The social identity theory emphasizes that membership in a group leads to the strengthening of personality and the formation of a kind of personal classification. According to this theory, the distinction between in-group and out-group is the basis for the formation of social (professional) identity ( 32 ).

Self-image is probably one of the most important elements of individual identity and self-esteem ( 33 ).

*Professional self-image* is, in fact, a description of one's self in relation to membership in the professional community. This description is often based on the general principles governing professional behavior, as well as one's perception of how being judged by others ( 26 ).

Trough continual experiences of socialization, in particular culture of profession, and with improving students' performance in relation to patients, the sense of self-worth increases, and therefore the students' self-image gradually approaches the professional image of role models. Similarly, group behavior, in the process of professional identity formation, gradually leads to a *sense of belonging* to group members, and *sense of distinction* from people outside the group ( 34 ).

***Evaluative component*** of affective realm which consists of two parts of professional *self-confidence*, and *self-esteem*, refers to one's self-assessment as a professional (physician), and depends on “comparison with others”, and “the judgment by others”. In other words, professional self-esteem is the result of a balance between ideal and real professional self-image ( 26 ).

***Normative component*** also includes *role perception, attitude toward profession, comfort in professional team, degrees of work centrality, and job satisfaction.* Role perception is, in fact, understanding the various aspects of the profession and its responsibilities ( 35 ). Research shows that people's perceptions and attitudes toward their professions can affect how they perform. The more positive these attitudes are, the more desirable and effective one's performance in the profession will be ( 36 ). Better performance facilitates professional identity formation by increasing self-confidence and will have consequences such as comfort in the professional team as well as job satisfaction ( 25 ).

***Prospective component*** includes *future job perspective*, and refers to people's expectations of developing their future careers. During the clinical years, students assess their suitability for work in various specialties by imagining themselves in related roles, and set their future career goals based on their final choices ( 16 ).

**Volitive Realm:** According to Haggard and Lau ( 37 ), volition is the human capacity to take action on the basis of inner decision and motivation, and is, in fact, “the link between thought and action”. Volitive realm of the psychological dimension of professional identity includes two main components: *job motivation and desire for professional activity.*

Medical education is a long and difficult process and requires high motivation (intrinsic motivation) to learn, academic achievement and professional identity development. Satisfaction with the three basic psychological needs is essential to stimulate and maintain this inner motivation: autonomy, competence, and relatedness ( 38 ).

The process of professional socialization can influence the motivation and desire to work in the medical profession.

### Social Dimension

The social dimension of professional identity in this model includes the communicative, cohesive, and operative realms.

**Communicative Realm:** Communication behaviors are the most obvious means by which the physician’s identity formation can be interpreted. Through communicative practices, new members, learn professional culture, and develop, maintain, and then reproduce medical ideology ( 39 ). Both verbal and nonverbal communication and the messages that students receive through them play a role in the development of their social (professional) identity ( 40 ). Through communication, students learn the meaning of symbols, jargons, and behavioral principles of group members and gradually become professional members in that community ( 41 ).

**Cohesive Realm:** The cohesive realm of the social dimension of professional identity consists of the following components: *entitativity, coherence, wholeness, acceptance by professional group, reciprocal trust, reciprocal commitment, and degrees of loyalty.*

*Entitativity* explains the distinction between real groups with similar goal or behavior from groups that are the result of mere aggregation of people. In other words, entitativity is the perception of a group as a single entity, distinct from its individual members ( 42 ). 

 Group coherence refers to the solidarity, and power of positive interaction between members of a group ( 43 ).

In other words, coherence is a kind of mutual attraction between the members of a group, which is due to the pursuit of common needs and goals that will lead to their satisfaction ( 43 ). 

Acceptance by the professional group is one of the most important components of the coherence realm, and requires learning the language and culture of the profession and acquiring and mastering common skills, which leads to developing effective communication with professional members ( 44 ). To build and maintain effective relationships between members of a professional team, it is important to have mutual trust ( 45 ). According to Cruess, Cruess, and Steinert ( 19 ) trust, and mutual respect are recognized as essential components of a community of practice, and the professional identity formation of its members.

Professional commitment is the desire to be part of a group, which is the result of work experience that meets one's needs for membership in that group. It seems that the experience of feeling comfortable in a professional role, and the sense of competence in performing tasks related to the role, are directly related to professional commitment ( 46 ).

By acquiring the professional competence, and going through the stages of socialization, students are gradually accepted into the professional community and feel comfortable among team members, thus committed to the goals and members of the profession.

**Operative Realm:** The last part of social dimension of professional identity consists of *involvement, engagement, effective professional performance and professional behavior.*

Students' conscious and semi-conscious behaviors from the beginning of their entry into the community of practice include observing the behaviors and relationships of group members, especially role models, and observing how they do things, gathering information about role expectations, and searching feedback, all influencing the formation of the social dimension of professional identity and the achievement of effective professional performance ( 4 ).

In addition to developing skills, students must learn professional behaviors that facilitate successful interaction with patients, their families, and colleagues. These behaviors reflect the student's initiative, time management skills, self-directed learning ability, and individual and organizational skills, and can be an indicator of a student's ability to perform professionally ( 47 ). During the socialization process, professional values are passed on to newcomers through socially agreed-upon professional behaviors to develop professional identity ( 48 ). 

## Discussion

The PMPI model presented in this study is a static structural model that depicts the structure of professional identity from both internal (psychological) and external (social) dimensions. The advantage of this model over other existing models is its comprehensive approach to the concept of professional identity.

Among the existing models in this field, some have focused on the factors influencing this process ( 2 - 4 , 11 ), some have prescribed interventions to improve it without providing conceptual bases ( 14 ), and some of these models have only examined this process from one dimension. For example, in Perez's model of professional socialization ( 13 ), the focus is only on the cognitive and psychological dimensions of professional socialization, whereas Weidman ( 3 ), and Weidman, Twale, and Stein ( 4 ) in their proposed models, deal only with the non-cognitive aspects of professional socialization and the social dimension of professional identity.

Some of these models also refer to some components of the two psychological and social dimensions of professional identity but in a non-structured way. For example, in the psychological dimension, Cruess et al. ( 11 ) refer to components such as “learning the (professional) language”, and “learning to live with ambiguity”; Miller ( 5 ), Cruess et al. ( 11 ), and Devenish, Clark, and Fleming ( 12 ) have proposed coping with the hierarchy of power; and Edens ( 2 ), Miller ( 5 ), Cruess et al. ( 11 ), Perez ( 13 ), and Byram ( 16 ) have mentioned values, attitudes, and professional culture all of which can be considered as cognitive components of professional identity. In another model, Byram ( 16 ) refers to the professional self-image, and sense of belonging to the profession that can be considered as descriptive components of the psychological dimension of professional identity. In addition, self-confidence in Devenish, Clark, and Fleming model ( 12 ) can be considered an evaluative component of psychological dimension of professional identity. 

Similarly, a number of elements of the social dimension of professional identity can be found in some of the models studied. For example, interaction and participation ( 3 - 5 , 11 , 13 - 14 ), role play ( 11 , 16 ), acceptance ( 11 , 12 , 16 ), and performance ( 14 , 16 ). 

Although all of these models have been able to help illuminate the hidden angles of this process, it seems that in order to deeply understand the process of professional formation, the dimensions and components of the concept of professional identity must be first properly displayed. 

By providing a comprehensive and in-depth view of the structure of professional identity, PMPI can be the basis for further research to draw a detailed roadmap and determine milestones in the development of each component of professional identity, as well as identify the factors influencing their development.

## Conclusion

In this study, based on a comprehensive review of existing models of professional identity, and analysis of each of the nine selected models, first the dimensions of professional identity and their components were extracted and then based on them, a static structural model for professional identity was developed. One advantage of this model is demonstration of dimensions, components and structure of the concept of professional identity, which is not yet available in the relevant literature.

In addition to clarifying the concept of professional identity, this model provides an opportunity to examine how the dimensions and components of this concept evolve over time, during the process of professional socialization, a development model for the formation of professional identity, which our research team is working on. This will provide an opportunity to monitor the evolution of professional identity during the socialization process, and provide appropriate interventions to facilitate and accelerate it.

### Limitations

This model is designed based on review and analysis of existing models and needs to be tested with empirical evaluations.

## References

[1] Cooke M, Irby DM, O'Brien BC (2010). Educating physicians: a call for reform of medical school and residency.

[2] Edens GE (1987). Professional Socialization in Nursing.

[3] Weidman J ( 1989). Undergraduate socialization: A conceptual approach. Higher education: Handbook of theory and research.

[4] Weidman JC, Twale DJ, Stein EL (2001). Socialization of Graduate and Professional Students in Higher Education: A Perilous Passage? ASHE-ERIC Higher Education Report.

[5] Miller SE ( 2010). A conceptual framework for the professional socialization of social workers. Journal of Human Behavior in the Social Environment.

[6] Monrouxe LV ( 2010). Identity, identification and medical education: why should we care?. Med Educ.

[7] Monrouxe LV, Rees CE, Hu W ( 2011). Differences in medical students’ explicit discourses of professionalism: acting, representing, becoming. Med Educ.

[8] Weaver R, Peters K, Koch J, Wilson I ( 2011). ‘Part of the team’: professional identity and social exclusivity in medical students. Med Educ.

[9] Jarvis-Selinger S, Pratt DD, Regehr G ( 2012). Competency is not enough: integrating identity formation into the medical education discourse. Acad Med.

[10] Burford B ( 2012). Group processes in medical education: learning from social identity theory. Med Educ.

[11] Cruess RL, Cruess SR, Boudreau JD, Snell L, Steinert Y ( 2015). A schematic representation of the professional identity formation and socialization of medical students and residents: a guide for medical educators. Acad Med.

[12] Devenish A, Clark M, Fleming M ( 2016). Experiences in becoming a paramedic: the professional socialization of university qualified paramedics. Creative Education.

[13] Perez RJ ( 2016). A conceptual model of professional socialization within student affairs graduate preparation programs. Journal for the Study of Postsecondary and Tertiary Education.

[14] Atherley AE, Hambleton IR, Unwin N, George C, Lashley PM, Taylor CG ( 2016). Exploring the transition of undergraduate medical students into a clinical clerkship using organizational socialization theory. Perspectives on medical education.

[15] Long DN (2017). Out of the silo: A qualitative study of paramedic transition to a specialist role in community paramedicine.

[16] Byram JN (2017). The professionalization of medical students: a longitudinal analysis of professional identity formation and professionalism perceptions in second and third year medical students 2017 [Thesis].

[17] Ibarra H ( 1999). Provisional selves: Experimenting with image and identity in professional adaptation. Administrative science quarterly.

[18] Dukerich JM ( 2001). Role transitions in organizational life: an identity-based perspective. The Academy of Management Review.

[19] Cruess SR, Cruess RL, Steinert Y ( 2019). Supporting the development of a professional identity: general principles. Med Teach.

[20] Kegan R (1982). The evolving self.

[21] Cruess RL, Cruess SR, Steinert Y ( 2016). Amending Miller’s pyramid to include professional identity formation. Acad Med.

[22] Cheryan S, Plaut VC, Handron C, Hudson L ( 2013). The stereotypical computer scientist: Gendered media representations as a barrier to inclusion for women. Sex roles.

[23] Bentley SV, Peters K, Haslam SA, Greenaway KH ( 2019). Construction at work: Multiple identities scaffold professional identity development in academia. Frontiers in psychology.

[24] Sadeghi Avval Shahr  H, Yazdani S, Afshar L ( 2019). Professional socialization: an analytical definition. Journal of medical ethics and history of medicine.

[25] Walker LO, Avant KC (2005). Strategies for theory construction in nursing.

[26] Kelchtermans G ( 1993). Getting the story, understanding the lives: From career stories to teachers' professional development. Teaching and teacher education.

[27] Kwon U, Beatty SE, Lueg JE ( 2000). Organizational values, work norms, and relational role behaviours: An empirical retail assessment. The International Review of Retail, Distribution and Consumer Research.

[28] Frankel MS ( 1989). Professional codes: Why, how, and with what impact?. Journal of business ethics.

[29] Kenny NP, Mann KV, MacLeod H ( 2003). Role modeling in physicians’ professional formation: reconsidering an essential but untapped educational strategy. Acad Med.

[30] Beagan BL ( 2001). “Even if I don't know what I'm doing I can make it look like I know what I'm doing”: becoming a doctor in the 1990s. Canadian Review of Sociology/Revue canadienne de sociologie.

[31] Lempp H, Seale C ( 2004). The hidden curriculum in undergraduate medical education: qualitative study of medical students' perceptions of teaching. BMJ.

[32] Turner JH, Turner PR (1978). The structure of sociological theory.

[33] Clearfield SM ( 1977). Professional self-image of the social worker: Implications for social work education. Journal of Education for Social Work.

[34] Handler EO (1970). The Professional Self Image and the Attributes of a Profession: An Exploratory Study of the Preschool Teacher.

[35] Eggen P, Kauchak D (2011). Strategies and models for teachers: Teaching content and thinking skills.

[36] Cristina-Corina B, Valerica A ( 2012). Teachers’ perceptions and attitudes towards professional activity. Procedia-Social and Behavioral Sciences.

[37] Haggard P, Lau H ( 2013). What is volition?. Exp Brain Res.

[38] Kunanitthaworn N, Wongpakaran T, Wongpakaran N, Paiboonsithiwong S, Songtrijuck N, Kuntawong P, et al ( 2018). Factors associated with motivation in medical education: a path analysis. BMC medical education.

[39] Apker J, Eggly S ( 2004). Communicating professional identity in medical socialization: considering the ideological discourse of morning report. Qualitative health research.

[40] Messersmith AS (2008). Becoming a nurse: The role of communication in professional socialization.

[41] Barnett GA, Carson DL (1983). The Role of Communication in the Professional Socialization Process [Thesis].

[42] Forsyth DR (2010). Group dynamics.

[43] Hogg MA ( 1993). Group cohesiveness: A critical review and some new directions. European review of social psychology.

[44] Meyer JP, Allen NJ, Smith CA ( 1993). Commitment to organizations and occupations: Extension and test of a three-component conceptualization. Journal of applied psychology.

[45] Kramer RM ( 1999). Trust and distrust in organizations: Emerging perspectives, enduring questions. Annual review of psychology.

[46] Meyer JP, Stanley DJ, Herscovitch L, Topolnytsky L ( 2002). Affective, continuance, and normative commitment to the organization: A meta-analysis of antecedents, correlates, and consequences. Journal of vocational behavior.

[47] Koenig K, Johnson C, Morano CK, Ducette JP ( 2003). Development and validation of a professional behavior assessment. Journal of Allied Health.

[48] Richardson B, Lindquist I, Engardt M, Aitman C ( 2002). Professional socialization: students' expectations of being a physiotherapist. Med Teach.

